# Continuous supplementary tactile feedback can be applied (and then removed) to enhance precision manipulation

**DOI:** 10.1186/s12984-020-00736-9

**Published:** 2020-08-28

**Authors:** Leonardo Cappello, Waleed Alghilan, Massimiliano Gabardi, Daniele Leonardis, Michele Barsotti, Antonio Frisoli, Christian Cipriani

**Affiliations:** 1 Scuola Superiore Sant’Anna, The BioRobotics Institute, Pisa, Italy; 2 Scuola Superiore Sant’Anna, Department of Excellence in Robotics & AI, Pisa, Italy; 3 Scuola Superiore Sant’Anna, TeCIP Institute, PERCRO Laboratory, Pisa, Italy

**Keywords:** Sensory feedback, Tactile feedback, Feedforward control, Haptics, Sensorimotor control

## Abstract

**Background:**

Human sensorimotor control of dexterous manipulation relies on afferent sensory signals. Explicit tactile feedback is generally not available to prosthetic hand users, who have to rely on incidental information sources to partly close the control loop, resulting in suboptimal performance and manipulation difficulty. Recent studies on non-invasive supplementary sensory feedback indicated that time-discrete vibrational feedback delivered upon relevant mechanical events outperforms continuous tactile feedback. However, we hypothesize that continuous tactile feedback can be more effective in non-routine manipulation tasks (i.e., tasks where the grip force is modified reactively in response to the sensory feedback due to the unpredictable behavior of the manipulated object, such as picking and holding a virtual fragile object) if delivered to highly sensitive areas. We further hypothesize that this continuous tactile feedback is not necessary during all the duration of the manipulation task, since adaptation occurs.

**Methods:**

We investigated the effectiveness of continuous tactile feedback in precision manipulation, together with a new sensory feedback policy, where the continuous tactile feedback is gradually removed when the grasp reaches a steady state (namely, transient tactile feedback). We carried out an experiment in a virtual-reality setting with custom tactile feedback devices, which can apply continuous pressure and vibrations, attached to the thumb and index finger. We enrolled 24 healthy participants and instructed them to pick and hold a fragile virtual cube without breaking it. We compared their manipulation performance when using four different sensory feedback methods, i.e., no tactile feedback, discrete vibrations, continuous tactile feedback, and transient tactile feedback. The latter consisted of gradually removing the continuous feedback in the static phase of the grasp.

**Results:**

Continuous tactile feedback leads to a significantly larger number of successful trials than discrete vibrational cues and no feedback conditions, yet the gradual removal of the continuous feedback yields to comparable outcomes. Moreover, the participants preferred the continuous stimuli over the vibrational cues and the removal in the static phase did not significantly impact their appreciation of the continuous tactile feedback.

**Conclusions:**

These results advocate for the use of continuous supplementary tactile feedback for fine manipulation control and indicate that it can seamlessly be removed in the static phase of the grasp, possibly due to the mechanism of sensory adaptation. This encourages the development of energy-efficient supplementary feedback devices for prosthetic and telemanipulation applications, where encumbrance and power consumption are burdensome constraints.

## Background

The marvelous complexity of human dexterity appears evident when we think about the wide variety of shapes and sizes of objects that we can grasp, the complex tasks that we perform with our hands, and our ability to adjust grasp types in response to changing task needs. This is not only due to the sophisticated physical structure of our hands but also to our control capabilities. It is established that these control capabilities rely on the sensory signals originated during contact between digits and objects, which are used by unimpaired individuals to represent the actions in the central nervous system (CNS) through internal models [1, 2]. Therefore, when the cutaneous feedback is missing, the internal models underlying the anticipatory control mechanisms are updated with poor or erroneous sensory inputs, resulting in inaccuracy of the model output, and ultimately in a deteriorated coordination. This is supported by the evident decline of force coordination during manipulation that follows anesthesia [3], and by the degradation of the grip force adjustments in response to accidental slips and of the grip force responses to unpredictable loads after anesthetic block [4].

Despite the acceptable level of motor function that the use of active prostheses can provide after the amputation of a hand, restoring the sensory function is not a trivial task. Indeed, sensory restoration is a major research topic in the field of prosthetics, both with invasive and non-invasive techniques [5–7]. Artificial supplementary sensory feedback (SSF) can be delivered to the prosthetic users in the same sensory modality (e.g., tactile stimuli corresponding to tactile events) – commonly referred as modality matched SSF – or in different modalities (e.g., auditory stimuli corresponding to tactile events). It can moreover be rendered in a continuous fashion (e.g., a pressure proportional to the contact force) or only correspondingly to certain discrete events (e.g., a short vibration) [6]. Although it is intuitive to argue that the prosthetic users would benefit from SSF [5], up to date the attempts in closing the control loop with modality-matched continuous SSF produced limited, yet controversial results [8–10].

Heretofore, we identified several reasons to the lack of successful results [11–13]. Firstly, the continuous SSF may have larger variance (e.g., due to noise or low sampling precision in the force sensors of the feedback system) with respect to visual feedback, causing the former to be disregarded by the CNS in favor of the visual cues that the prosthetic users get by looking at the hand [14]. In fact, it is known that for object manipulation the CNS may switch to alternative sources of afferent information when cutaneous feedback is lost [15], and that vision can compensate for the permanent loss of proprioception to update the central representation of limb dynamics [16]. Secondly, unreliable and imprecise control of the prosthesis [17], introduced by the intrinsic limitations of surface EMG control (i.e., its susceptibility to environmental noise and the unavoidable delays introduced by signal acquisition and classification), and uncertainties in the feedforward path [18], such as the delays introduced by the mechanical hardware in the loop, might contribute to limiting the participants’ ability to exploit the continuous SSF [13]. Thirdly, it is known that the tactile stimuli take 14–28 ms to reach the CNS in able-bodied individuals [19], and it is suggested that artificial SSF should be delivered in a fraction of that time to promote the effective use of the sensory information [6]. Delays in the SSF devices may, therefore, hinder their functionality. Fourthly, non-invasive continuous SSF is inevitably delivered to the forearm, the arm, or the chest of the amputees; however, these areas are less innervated than the glabrous skin of the hand and therefore less sensitive to tactile stimuli [20], and they are not normally engaged in the manipulation tasks. Their recruitment for manipulation tasks ultimately leads to a cognitive burden [21]. Finally, the mechanism of sensory adaptation to continuous haptic stimuli [22, 23] may limit the effectiveness of the SSF. We further believe that another limiting factor to the spread of tactile feedback devices is the burden that they impose in terms of weight, energy consumption and bulkiness. In fact, SSF devices feature electric motors that need to be continuously powered or non-backdrivable systems that decrease the efficiency of the transmission and add extra weight to the already burdensome prosthesis, which would also require larger batteries to be powered.

Conversely, recent evidences showed that time-discrete short-lasting vibrotactile feedback stimuli delivered correspondingly to the most relevant mechanical events of manipulation (i.e., object contact, liftoff, replace, release), based on the *Discrete Event-driven Sensory feedback Control* policy [24] (hereby referred as DESC feedback), are integrated by users controlling a prosthetic hand if these stimuli are related to predictable events that are delivered with low latency [11, 12]. In other words, the DESC feedback is effective if the task and the manipulated object are represented with an internal feedforward model [1, 25]. Interestingly, DESC vibrotactile feedback can easily be implemented into prosthetic devices thanks to the very low power consumption required, and the availability of miniature vibrators [12]. Nonetheless, incidental feedback (i.e., all the feedback sources that are implicitly available to the prosthesis users – as opposed to the explicit feedback provided by the SSF devices – e.g., socket vibration, muscle contraction, motor sound, proprioception of the residual limb) and myoelectric control together with visual feedback can per se provide amputees with enough information to learn the inverse dynamics of the prosthetic system to a certain extent, contributing to a partial prediction of the grasping force that they apply [26].

However, for its nature, DESC feedback provides the users with less sensory information than the continuous SSF. Moreover, we hypothesized that if the incidental tactile feedback is inadequate or absent, or if the SSF is delayed or delivered to insufficiently sensitive sites not normally engaged in the manipulation tasks, the CNS may not learn the inverse dynamics of the motor task, as these signals might be subject to sensory cancellation [27]. As a result, the internal model of the task may be erroneous and, consequently, the DESC feedback stimuli could result ineffective in the overall task performance since the CNS cannot relate them to predictable events.

Therefore, in this work we aimed at further investigating the potential of continuous SSF, attempting at overcoming its aforementioned limitations**.** With this aim, we hypothesized that if the continuous SSF is delivered to highly sensitive sites (such as the fingertips or areas treated with Targeted Sensory Reinnervation [28], referred ad TSR) it can be employed by the users without cognitive burden. TSR is a recent ground-breaking surgical technique that provides amputees with near normal hand sensation on skin areas of the residual limb through peripheral nerve rerouting, which might be the cornerstone for translation of continuous SSF techniques to clinical practice. Finally, it is known that all afferent tactile fibers are characterized by a different adaptation time – i.e., a decline in the discharge of a receptor while the stimulus is unchanging [19, 24, 29, 30] – making prolonged continuous static feedback progressively disregarded by the CNS. This adaptation time ranges from fractions of a second to several seconds depending on the tactile afferents, which are in fact classified into fast-adapting and slow-adapting types [19]. We suggest that this mechanism, known as sensory adaptation, could be advantageously exploited to mask the gradual removal of the continuous SSF in the static phases of the manipulation tasks without significantly impacting on the grasp performance. This would allow saving a considerable amount of power during manipulation, which is a major concern in prosthetics.

Scope of this work was twofold: to investigate i) whether the continuous SSF delivered to highly sensitive areas of the skin can be used to improve the manipulation performance for tasks that require fine control of grasp force in absence of incidental feedback other than vision, and ii) whether it is possible to limit the application of the continuous SSF only to certain phases of the grasp without degrading the task performance.

In attempt to answer the abovementioned questions, we conducted experiments with healthy participants who were trained to manipulate a fragile cube in a virtual reality environment. The setup created a condition where incidental feedback was minimized. The position of the participants’ digits was tracked with an optical system. Custom miniature wide-bandwidth linear actuators were used to convey different modalities of tactile feedback in the form of normal force applied to the fingertips of the index and the thumb.

## Materials and methods

### Participants

Twenty-four right-handed participants (age range: 22–36 years, 15 males) with no known neurological history were enrolled in this study. The study was performed in accordance with the Declaration of Helsinki and approved by the Ethics Committee of the Scuola Superiore Sant’Anna (approval number 2/2017). All participants gave written informed consent before participating in the study.

### Apparatus

#### Haptic thimble device

**Fig. 1:**
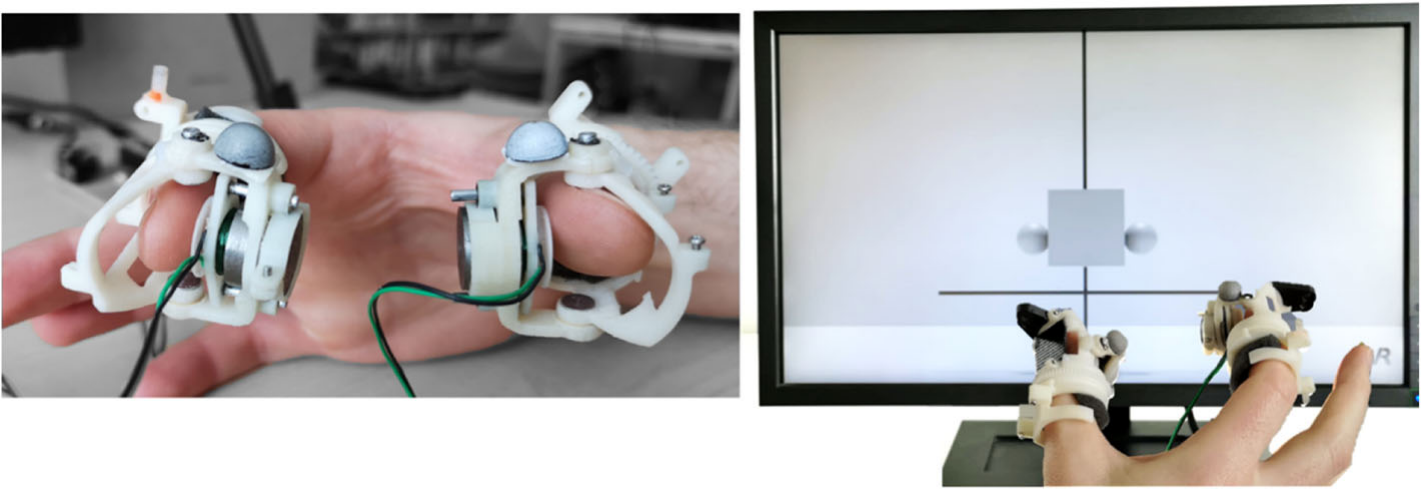
The Haptic Thimble devices with linear high-bandwidth linear actuators for haptic rendering and with reflective markers mounted on the upper side for tracking purposes (left), and the virtual reality environment (right).

To deliver normal contact forces and vibrations to the digits of the participants, two custom fingertip feedback devices were used, namely, the Haptic Thimbles, adapted from Gabardi et al., 2016 [31] (Fig. 1), consisting of custom-built linear actuators driven by a microcontroller (Teensy 3.2, Pjrc.com LLC, USA). They featured a prismatic joint actuated by a voice coil, guided by two miniature shafts sliding with low friction bushes, with a maximum stroke of 4 mm. The terminal part of the prismatic joint is in contact with the fingertips through a 3D-printed plate, which ultimately delivers the feedback stimuli. They are able to render both continuous forces of up to 1 N and vibratory stimuli with a frequency of up to 250 Hz. Their small size (66x35x38 mm) and light weight (22 g) did not obstruct the natural motion of the fingers and did not significantly fatigue the users during the experiments.

#### Optical tracking system and virtual environment

An optical tracking system (OptiTrack V120 Trio, Motive, USA) was used to capture the absolute position in the 3D space of two reflective markers fixed on the Haptic Thimbles, with a frequency of 120 Hz. These position data were streamed to a virtual reality environment (Fig. 1) developed on XVR framework [32] and running on a desktop computer. The virtual scene rendered the position of the fingertips with two spheres with 10 mm diameter, colored in light grey, and it also comprised a floor plane, a virtual cube laying on it with dimensions of 50x50x50 mm, and a horizontal target line placed 100 mm above the floor plane as in Leonardis et al., 2017 [32]. The relative distance of the fingertips (namely, interdigit distance) corresponded in the virtual environment to a symmetrical translation of the two spheres along the horizontal axis, while the elevation of the central point of their distance corresponded to a vertical translation of the spheres, both with a 1:1 mapping from the real world to the virtual workspace. A virtual sliding joint, represented as a vertical line in the virtual reality, constrained all the degrees of freedom of the cube except for the vertical translation. The point of view was coherent with the body pose of the participant with the cube in front of them. The dimensions of the virtual workspace corresponded to their actual size on the monitor.

#### Dynamic model

The dynamics of the virtual scene was simulated within the XVR environment. The virtual cube was modeled as a linear elastic object with stiffness *k* equal to 0.125 N/mm. When the measured interdigit distance *d* was lower than 50 mm, the spheres began to interpenetrate the cube and the contact force *F*_*c*_ linearly increased, as described in Eq. (1):

1 $$ {F}_C=\left\{\begin{array}{c}0\  if\ d>50 mm\\ {}k\left(50-d\right) if\ d<50 mm.\end{array}\right. $$

The virtual mass of the cube was set to 30 g, and the friction coefficient between the spheres and the cube was set to 1. This meant that if the contact force was below the slip threshold of 0.15 N (that corresponds to 1.2 mm of interpenetration), the cube began to slip. If the contact force was above this value, the cube moved together with the spheres along the vertical direction. Furthermore, a breaking threshold equal to 0.75 N (corresponding to 6 mm of interpenetration) was set on the virtual cube to simulate it fragile.

### Experimental procedure

**Fig. 2:**
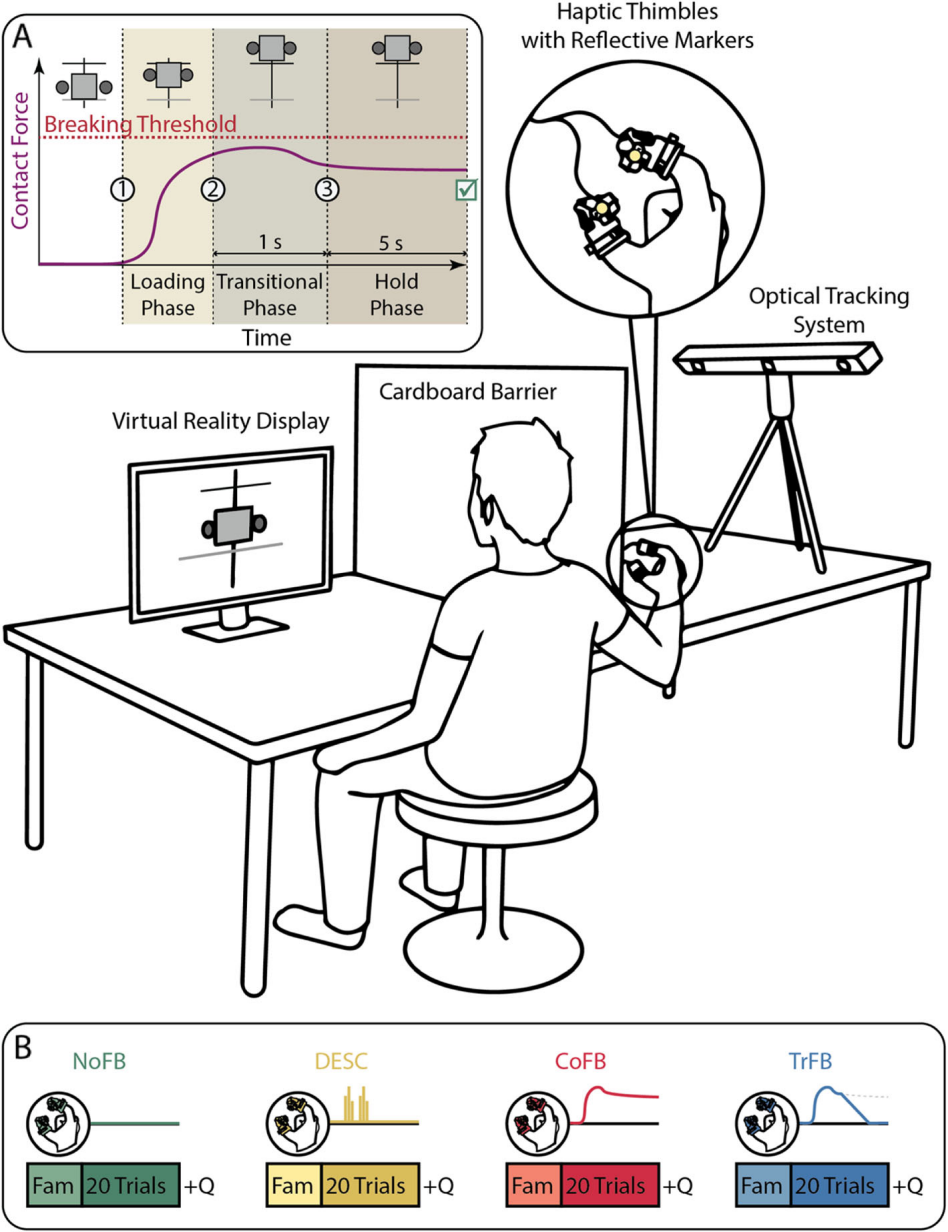
The experimental setup, where the subject sits at a desk and performs a virtual reality pick-and-lift task while wearing the Haptic Thimble device (the position of the participant’s arm is exaggerated for clarity purpose). In A) a typical successful trial is shown, where the object is approached and lifted above the target line (black solid line), while the contact force exerted (purple solid line) is below the breaking threshold (red dashed line). Vertical dashed lines represent the separations between the phases of the grasp. In B) the four experimental conditions are exemplified: for each of them, a familiarization (Fam) phase precedes the execution of 20 trials of the task, while a distinct tactile feedback is provided. A questionnaire (Q) follows each condition.

The participants took part in a single-day experimental session, where they were asked to sit at a table and wear the Haptic Thimbles on the pads of the thumb and the index finger. A monitor placed in front of them displayed the virtual scene, while a cardboard barrier prevented them from seeing their hand and the Haptic Thimbles (Fig. 2). The participants were instructed to repeatedly complete a trial consisting of smoothly grasping the virtual cube and holding it above the horizontal target line for 6 s as fast as they could without breaking it. The color of the cube turned from grey to red if it was broken (i.e., the contact force exceeded the breaking threshold) or slipped (i.e., the contact force was lower than the slip threshold), and it turned green upon successful execution of the task. Each trial terminated with one of the following results: i) success: the cube was correctly lifted from the reference floor and lifted above the target line for the required amount of time; ii) break failure: the cube was grasped with a contact force exceeding the breaking threshold; and iii) slip failure: the cube was lifted, but the contact force decreased and the cube slipped. At the end of each trial, whether it was completed with success or failure, the participants were instructed to open their digits and wait for the virtual scene to reset. Moreover, we divided the lifting task into three phases, adapted from Johansson and Edin, 1993 [24], marked by specific events: i) the loading phase, which starts at the onset of the contact and ends when the cube is lifted above the target line, ii) the transitional phase that follows the lift phase and ends 1 s after it, and iii) the hold phase, which covers the remainder of the manipulation task. We chose these phases since they are characterized by different behaviors of the contact force: in the first phase, the contact force is established and is dynamically adjusted until the object is lifted to the target position; in the second phase, the contact force is stabilized to a (almost) constant value; in the third phase, the grasp is stable and only subtle changes in the contact force are expected. The duration of the transitional phase was chosen assuming that 1 s after the liftoff was more than enough for the grasp to stabilize (i.e., no significant fluctuations of forces and position were noticeable) [33].

The virtual contact force was delivered to the participants according to four different conditions characterized by the following feedback modalities: i) no supplementary sensory feedback (**NoFB**), where only the visual feedback of the virtual reality was available to the participants, ii) discrete vibrational bursts of 100 ms at 70 Hz (selected to be perceivable by all the participants) upon contact and lift-off events according to the DESC model (**DESC**), iii) continuous tactile feedback (**CoFB**) equal to the contact force *F*_*C*_, and iv) transient feedback (**TrFB**), where the continuous tactile feedback was equal to the contact force during the loading and the transitional phase, and it was subsequently linearly decreased to zero in 3 s during the hold phase, as illustrated in Eqs. (3) and (3):

2 $$ {F}_{TrFB}=\lambda {F}_c $$

3 $$ \lambda =\left\{\begin{array}{c}1\  if\ 0<t<1\\ {}1-\frac{t-1}{3}\  if\ 1<t<4\\ {}0\  if\ t>4\ \end{array}\right. $$

where λ is the coefficient for linearly remove the continuous contact force in 3 s. All the haptic feedback stimuli (vibrational, continuous, and transient) were displayed to the participants by the Haptic Thimbles in the form of normal forces, evenly applied to the pads of the index finger and the thumb.

During the experimental sessions, the participants were instructed to complete 20 trials for each condition, for a total of 80 trials per session. A familiarization phase preceded each condition, where the participants could freely interact with the virtual cube and experience the feedback modality for about 2 min. To compensate for the possible bias introduced by the presentation order, a unique permutation of the four conditions was administered to each participant. Therefore, the number of the participants was chosen to test all the possible permutations of the four experimental conditions.

Finally, to assess and compare how the different feedback modalities were subjectively perceived by the participants, they were requested to fill out a questionnaire after every condition. Eight questions were asked (see Table 1), and the participants could answer with a number between − 3 and 3, where − 3 meant “not at all” and 3 meant “very much”.

**Table 1:** Questionnaire

Questions	
Q1	How much did you rely on the haptic feedback?
Q2	How much did you rely on the visual feedback?
Q3	How realistic was the haptic feedback?
Q4	How much were the haptic feedback and visual feedback congruent?
Q5	How easy was it to control the contact force?
Q6	How positive was your experience in general?
Q7	How was your confidence level with the haptic feedback?
Q8	How easy was it to accomplish the task?

### Data collection and statistical analysis

To estimate the participants’ manipulation performance during each condition and each phase of the grasp we used the following indexes: percentage of trials completed with success (success rate), percentage of trials failed due to breakage of the cube (breakage rate), and percentage of trials failed due to slippage of the cube (slippage rate). Moreover, we counted the percentage of trials completed with success during lifting (successful lift rate), i.e., from the onset of the liftoff to when the cube was lifted above the target line. Finally, we measured the duration of the loading phase (loading time). For each of the performance indexes and questionnaire marks, the difference between the four different feedback conditions was tested with a non-parametric Friedman test for repeated measures. In case of a significant outcome (*p* < 0.05), Bonferroni-corrected pair-wise multiple comparisons were performed using Wilcoxon tests.

Furthermore, the position data of the markers in the 3D space and the position data of the cube in the virtual environment were recorded and stored. Beyond kinematic information, these data allowed us extracting the interaction dynamics, since the virtual forces applied to the object are related to the position data by the virtual stiffness k (in the continuous and transient conditions) and the coefficient λ (in the transient condition). To identify significant fluctuations of the interdigit distance in the hold phase from the transitional phase, the single-trial baseline permutation statistical method for inference testing was used [34]. In particular, the finger position in the time interval of the transitional phase was chosen as the baseline for normalization. Then, for each participant and each condition, 500 baseline permutations (across both time and trials) were used for extracting the bootstrap confidence interval with a significance level of 0.05. The interdigit distance profile averaged across participants in each condition was considered statistically significant when it lied outside the 2.5% or 97.5% tails of the lower or upper confidence interval collected in that specific condition (bootstrap statistic, *p < 0.05*).

## Results

**Fig. 3:**
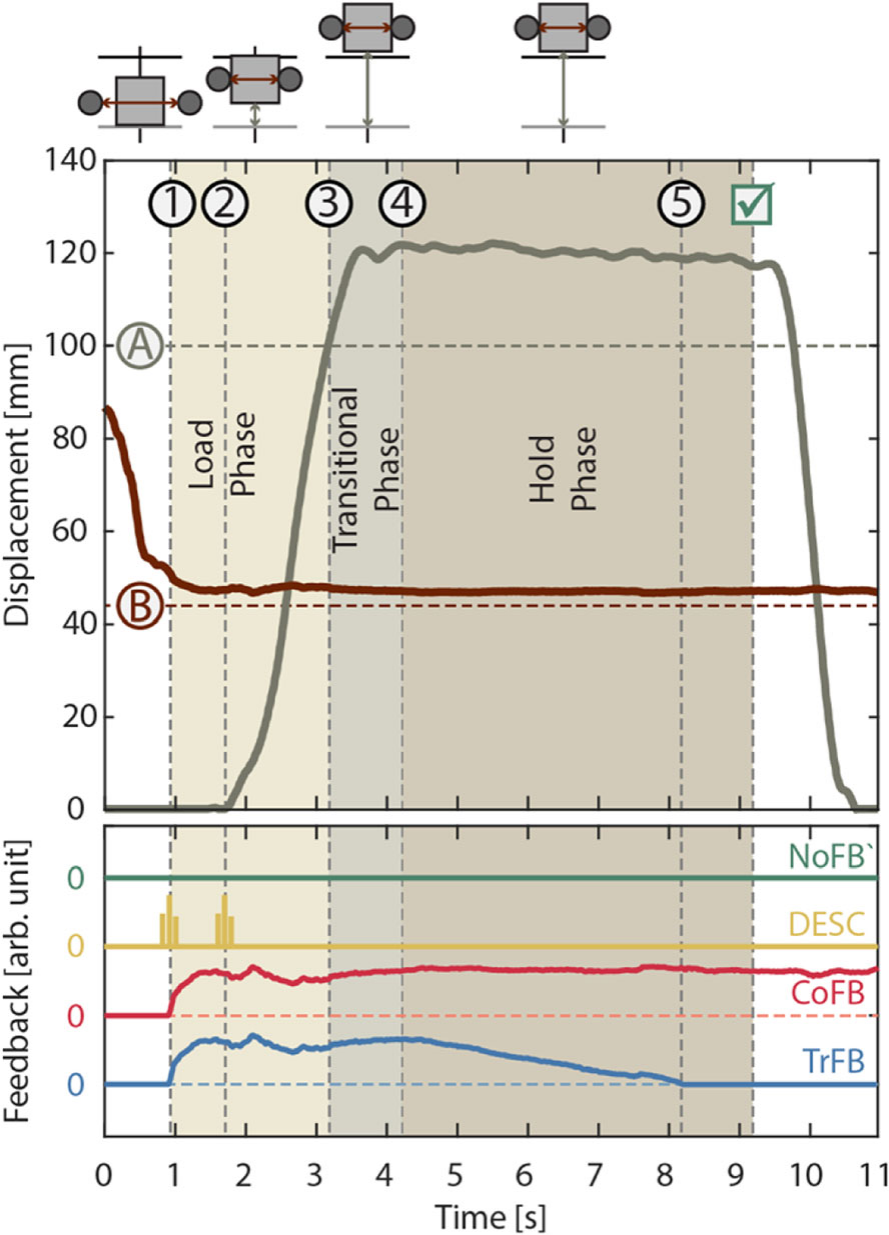
Representative successful trial, where the participant reduced the interdigit distance (dark red solid line) to grasp the object and lifted it (dark grey solid line) above the target line (dark grey dashed line A) without overcoming the breaking threshold (dark red dashed line B). The three phases of the grasp are depicted, together with the relevant events, such as: 1) object contact, 2) object lift-off, 3) object lift above the target line, 4) end of transitional phase, 5) termination of the transient feedback. In the panel below, with an arbitrary scale, a graphical representation of the four feedback modalities corresponding to the trial are reported. Notably, in the DESC modality short-lasting vibrations are delivered at contact and lift-off, in the continuous modality the tactile feedback is proportional to the contact force – as for Equation (1) - and the transient modality is equal to the continuous one except that after the transitional phase the tactile feedback is gradually removed.

**Fig. 4:**
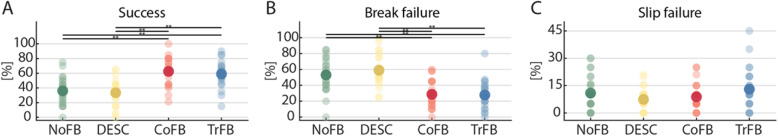
Overall performance indexes in the four different feedback conditions. Light dots represent single participants whereas solid dots represent the averaged value across participants. Significance of Bonferroni corrected Post-hoc test is shown (∗, *p* < 0.05; ∗∗, *p* < 0.01).

All the participants readily mastered the experimental task with the four feedback conditions (Fig. 3). The success rate proved significantly higher when the participants received the CoFB and the TrFB with respect to the NoFB and the DESC conditions (Friedman test: *Χ*^*2*^_*(3)*_ = 39.26, *p* < 0.001, followed by Bonferroni-corrected post-hoc analysis) (Fig. 4a). In particular, the participants achieved an average success rate of 62.5 and 59.0% under CoFB and TrFB conditions, respectively, whereas only 36.0 and 33.5% under the NoFB and the DESC conditions, respectively. Similar results were obtained from the analysis conducted on the percentage of broken cubes (*Χ*^*2*^_*(3)*_ = 43.67, *p* < 0.001), highlighting a significant improvement with the CoFB and TrFB with respect to the NoFB and DESC conditions (Fig. 4b). As regard to the percentage of cubes slipped away during the whole trial, no significant differences emerged across the four conditions (*Χ*^*2*^_*(3)*_ = 3.79, *p* = 0.29) (Fig. 4c). We can therefore observe a general increase in performance when continuous type feedback (TrFB and CoFB) is applied, with respect to both the no feedback (NoFB) and the discrete feedback (DESC) conditions.

**Fig. 5:**
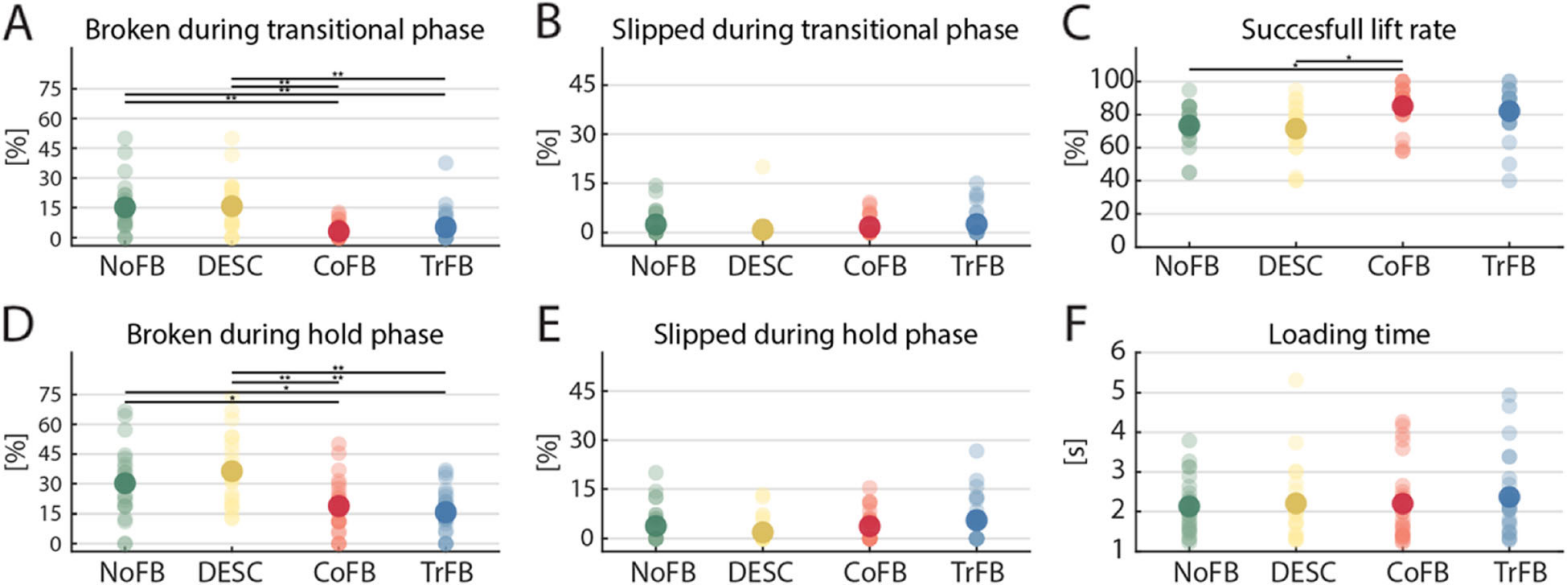
Performance indexes. Light dots represent single participants whereas solid dots represent the mean values across the participants. Significance of Bonferroni corrected Post-hoc test is shown (∗, *p* < 0.05; ∗∗, *p* < 0.01).

The percentages of broken and slipped cubes were analyzed separately for the time periods of the transitional phase and the hold phase (Fig. 5a,b,d,e). As regards the percentage of slipped cubes (Fig. 5b,e), no significant differences among the feedback conditions were found neither during the transitional phase (*Χ*^*2*^_*(3)*_ = 4.93, *p* = 0.18) nor during the hold phase (*Χ*^*2*^_*(3)*_ = 5.23, *p* = 0.16). Concerning the percentage of broken cubes, there was a statistically significant difference in the number of broken cubes depending on the type of sensory feedback, both in the transitional phase (*Χ*^*2*^_*(3)*_ = 30.50, *p* < 0.001) and in the hold phase (*Χ*^*2*^_*(3)*_ = 26.50, *p* < 0.001). Post-hoc analysis conducted on the percentage of broken cubes during the transitional phase revealed that when the participants received the CoFB and the TrFB they performed better compared to the NoFB and the DESC conditions. Regarding the percentage of broken cubes in the hold phase, it was found that the CoFB and the TrFB led to a smaller number of broken cubes with respect to both the NoFB and the DESC feedback (Fig. 5d).

The successful lifts (Fig. 5c) differed based on the sensory feedback (*Χ*^*2*^_*(3)*_ = 19.35, *p* < 0.001). In particular, post-hoc test highlighted a significant improvement under the CoFB condition (85.19%) with respect to the NoFB and DESC conditions (73.44% NoFB; 71.47% DESC). The TrFB produced a larger number of successful lifts (82.14% TrFB) than NoFB and DESC; however, this difference proved not significant. Moreover, we found that the different feedback modalities did not significantly affect the loading time (Fig. 5f), which was the same for all the conditions even though the participants were not constrained to accomplish the task within a specific time (NoFB = 1.96 s; DESC = 2.04 s; CoFB = 1.75 s; TrFB = 2.09 s; *Χ*^*2*^_*(3)*_ = 1.25, *p* = 0.74). Detailed results of the statistical analysis are reported for completeness in Table 2.

**Table 2:** Statistical test results (*, *p* < 0.005; **, *p* < 0.01)

Parameter	Friedman		Post-hoc test results				
	*Χ*^*2*^_*(3)*_	*p*		NoFB	DESC	CoFB	TrFB
Success	39.26	1.53E-08**	NoFB	-	-	-	-
			DESC	1	-	-	-
			CoFB	5.67E-04**	2.31E-04**	-	-
			TrFB	8.03E-04**	2.16E-04**	1	-
Break failure	43.67	1.77E-09**	NoFB	-	-	-	-
			DESC	1	-	-	-
			CoFB	1.96E-03**	1.22E-04**	-	-
			TrFB	1.31E-03**	1.08E-04**	1	-
Slip failure	3.79	2.9E-01	-	-	-	-	-
Broken tr.	30.50	1.09E-06**	NoFB	-	-	-	-
			DESC	1	-	-	-
			CoFB	1.93E-03**	4.44E-04**	-	-
			TrFB	2.55E-03**	7.82E-03**	1	-
Slipped tr.	4.93	1.8E-01	-	-	-	-	-
Broken hold	26.50	7.49E-06**	NoFB	-	-	-	-
			DESC	5.12E-01	-	-	-
			CoFB	1.29E-02*	4.83E-04**	-	-
			TrFB	1.41E-02*	1.22E-03**	1	-
Slipped hold	5.23	1.6E-01	-	-	-	-	-
Succ. lift rate	19.35	2.31E-04**	NoFB	-	-	-	-
			DESC	1	-	-	-
			CoFB	1.70E-02*	1.40E-02*	-	-
			TrFB	3.07E-01	6.14E-02	1	-
Loading time	1.25	7.4E-01	-	-	-	-	-

**Fig. 6:**
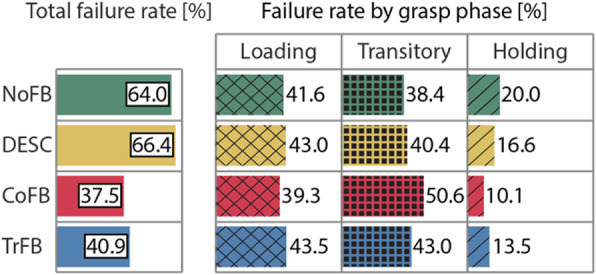
On the left, histogram with total relative failure rates for each feedback modality, computed as the sum of slip and broken failures for each phase for all the participants, divided by the total number of trials occurrences of failures of each condition. On the right, the relative failure rates divided by grasp phase, computed as the sum of slip and broken failures for each phase for all the participants, divided by the total occurrences of failures of each condition.

We also computed the relative occurrences of the failures (slip and broken) divided by phase, for each feedback modality for all the participants (Fig. 6, right). We observed a similar distribution of the failures across the modalities, with the larger number of failures (~ 80–90%) almost equally split between the loading and the transitional phases, regardless the difference between conditions in terms of total failure rates (Fig. 6, left).

**Fig. 7:**
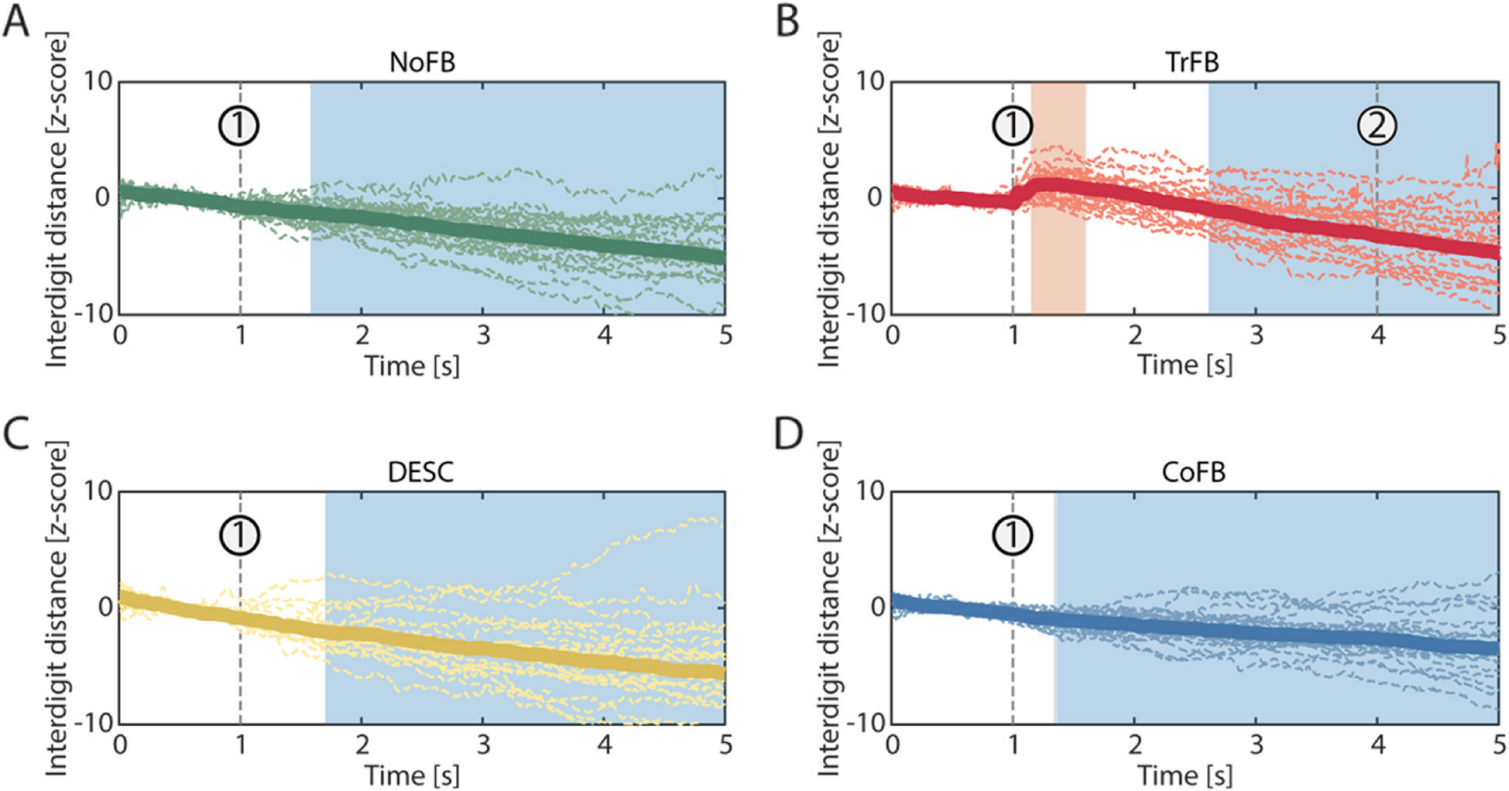
Interdigit distance in the four feedback conditions for the trials completed with success, during transitional (first second) and hold (last 4 seconds) phases. The sixth second of the hold phase has been disregarded since it was not relevant. Data are standardized over the transitional phase (first second) and reported in terms of z-score. Dashed lines represent the single participants, solid lines represent the mean value across the participants. Significant regions (permutation statistic, *p* < 0.05) are highlighted with a blue (for smaller distances) or red (for larger distances) background areas. The vertical dashed lines marked with 1 represent the beginning of the hold phase (i.e., the beginning of the feedback removal), while the vertical dashed line marked with 2 represents the instant when the transient feedback is equal to zero – see Equation (3).

The interdigit distance collected in the hold phase of the successful trials (Fig. 7) highlighted a tendency of the participants to close the fingers in all the feedback conditions (bootstrap statistics, *p < 0.05*), and this effect became significant in a time interval comprised between 1 and 2 s after the object was lifted above the target line for all but the TrFB conditions. Interestingly, in the TrFB condition, the interdigit distance increased for about 500 ms short after the beginning of the gradual removal of the sensory feedback, which became significant after about 100 ms after the transitional phase, to subsequently decrease afterwards.

**Fig. 8:**
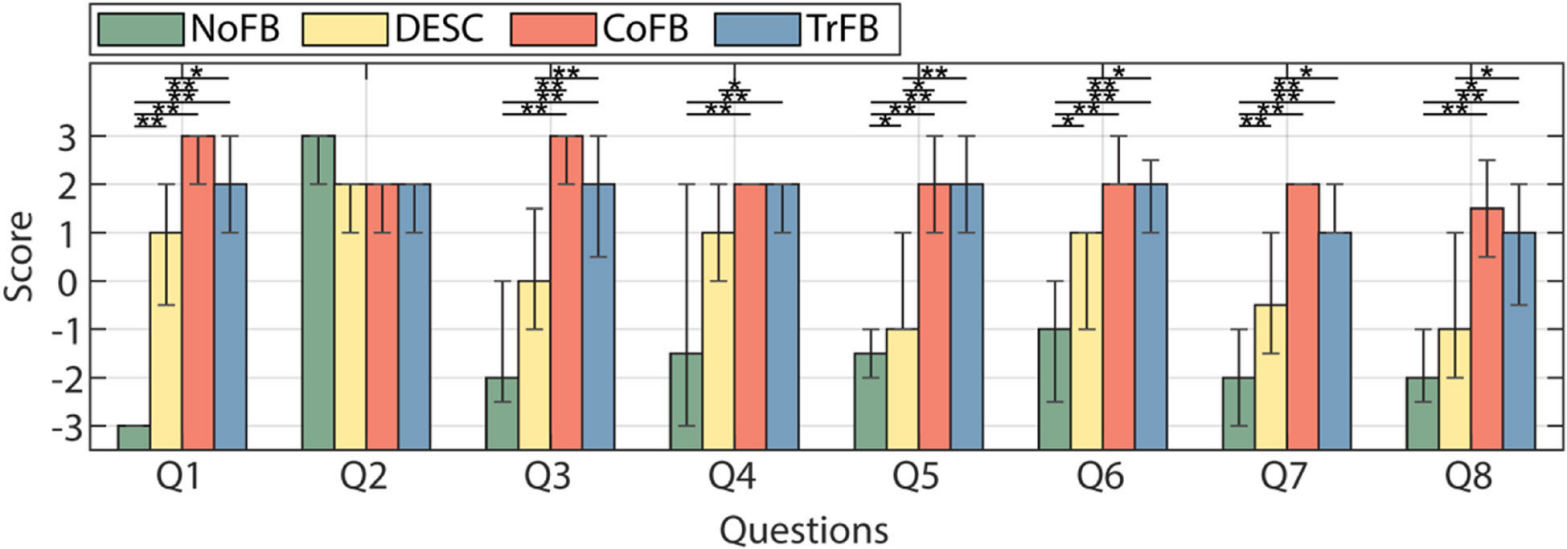
Answers to the experimental questionnaire. Height of the bars represents the median score whereas whiskers represent 25th and 75th percentiles. Asterisks mark Bonferroni corrected post-hoc significant differences (∗, *p* < 0.05; ∗∗, *p* < 0.01).

Finally, the subjective metrics indicated that the CoFB and TrFB conditions generally scored better than the other conditions and that they were not perceived differently by the users, evident by the lack of any significant difference among the responses to the questionnaire (Fig. 8). Notably, after they were interrogated by the experimenters about how they experienced the feedback, the participants reported that they noticed the continuous feedback changing during the TrFB condition, although they were not certain about this. CoFB and TrFB conditions generally scored better than the DESC condition, which scored significantly better than the NoFB condition in some of the metrics. In particular, the participants reported that they relied on the tactile feedback, with a significant preference towards the continuous stimuli (CoFB and TrFB) with respect to the DESC ones (Q1 - Fig. 8). In general, visual feedback was considered important for the execution of the task regardless of the feedback modality (Q2). The participants indicated that the CoFB and the TrFB conditions were both perceived as very realistic (Q3) and that they found an overall good congruency (Q4) between all the haptic feedback modalities (excluded the NoFB) and the visual feedback from the virtual scene. Although the DESC feedback was perceived as less realistic than the CoFB and TrFB conditions, it was perceived as somewhat realistic. Under the CoFB and TrFB conditions the participants perceived a great control over the contact force (Q5) and experienced a good confidence level (Q7). Conversely, the DESC condition was perceived less controllable and yielded a lower confidence level. Finally, the participants preferred the CoFB and the TrFB conditions to the NoFB and the DESC (Q6) and found it significantly more difficult to accomplish the task when receiving the latter two feedback modalities (Q8).

## Discussion

The aim of this study was to prove the possibility of employing continuous tactile feedback in precision tasks where incidental feedback was not available in order to promptly improve the manipulation performance. We also aimed at proving that in the static phase of the grasp it is possible to remove this feedback without deteriorating the manipulation performance. Our results confirmed the great potential of the transient tactile feedback. In fact, the participants needed minimal familiarization to employ this feedback policy proficiently.

The low success rate in the NoFB condition suggests that the virtual reality task posed a significant challenge to the participants in evaluating the physical properties of the object without tactile feedback. This is likely attributed to the lack of incidental information about the virtual cube [26], e.g. its inertial and frictional properties, the contact noise, etc., which prevented the predictive control from taking over. Furthermore, the visual feedback provided only indirect information about the motor task and the interactions with the virtual environment, which led to suboptimal performance and a larger percentage of task failures [19, 35]. Moreover, we can infer that the proprioceptive feedback naturally available to the participants (i.e. their joint position sense) was not enough to effectively close the control loop. The relatively long loading time (~ 2 s) suggests that the participants needed to wait for the sensory information to be conveyed to and processed by the CNS to plan the motor actions [25, 27]. This supports our hypothesis that the manipulation task was performed primarily relying on a *reactive* sensory feedback mechanism rather than on a *predictive* feedforward control [24]. In fact, the participants could not reliably employ and trust a forward model to accomplish the only apparently simple task without making mistakes (i.e., breaking the virtual object): similarly to operating a power tool [25], the task was not routine/stereotypical.

On the other hand, when the participants manipulated the virtual cube under the CoFB and TrFB conditions, they performed significantly better than with bare visual feedback. We can therefore deduce that the continuous feedback provided fundamental tactile information that people could effectively leverage to close the feedback control loop, and continuously regulate the grasp force. Receiving the continuous feedback did not add cognitive burden to the task. In fact, the participants employed the same time to lift the object above the target line for all the conditions, which may suggest that the continuous information was easily incorporated in the sensorimotor control.

Moreover, it is worth noting that in our non-routine task the DESC feedback yielded outcomes comparable to the visual only condition, and significantly lower than with the continuous feedback conditions (CoFB and TrFB). This outcome is in agreement with the DESC model, which posits that the feed-forward (predictive) control is effective only if the inverse dynamics of the task are well known, if the CNS effectively learned its internal model, and if it could relate discrete mechanical events to known phases of the task [24, 25]. Notably, these results are only apparently in contrast with our previous studies [11, 12], where in fact the tasks were stereotypical.

Interestingly, under the TrFB condition the manipulation performance did not deteriorate with respect to the CoFB. The mechanism of sensory adaptation may explain why these two conditions are substantially equivalent. They differed only in the hold phase of the grasp and, although the mechanoreceptors never completely stop firing in response to external prolonged stimuli [29, 30], the CNS may have disregarded the static tactile information provided by the CoFB condition during the hold phase after a short time, which was therefore unnecessary for motor control of manipulation.

From our results, we could speculate that in every phase of the task it is more useful to receive continuous type feedback (CoFB and TrFB), but the greatest difference in terms of relative reduction of broken failures from CoFB and TrFB to DESC and NoFB could be observed in the transitional phase (with a relative difference of ~ 70%), while during the hold phase this difference tended to reduce (with a relative difference of ~ 50%). Interestingly, during the loading phase, the TrFB condition did not produce significantly different outcomes with respect to the other conditions in terms of successful lifts, but we believe that this effect is produced by the relatively small sample size, and significance could have arisen if we tested a larger population. Moreover, we can observe that the majority of failures took place in the loading and in the transitional phases, further supporting our claim that receiving feedback in the hold phase is not crucial for manipulation.

The questionnaires are in line with the objective metrics of the study: under the TrFB and CoFB, the participants produced the best performances. These feedback conditions were perceived as substantially equivalent by the participants, supporting our hypothesis that the mechanism of sensory adaptation could be exploited to mask the gradual disappearing of the continuous feedback. Notably, the participants expressed a significant preference for the CoFB and the TrFB conditions, which represents an extremely valuable factor for feedback design. In details, it is worth noting that the removal of the feedback (TrFB condition) did not significantly impact the high perceived controllability and confidence level that the participants experienced under the CoFB. In fact, they both resulted in a good overall experience and ease to accomplish the task. Overall, we can speculate that people integrate SSF in non-routine manipulation tasks to perform them more comfortably and perceiving a lower cognitive burden. Finally, the great acceptance of the DESC feedback in the sensorimotor control is confirmed [11], albeit, continuous and transient feedback showed better acceptance in non-routine manipulation tasks.

The tendency of the interdigit distance to get smaller in the hold phase is well known: to maintain the fingers extended, a continuous (and fatiguing) contraction of the extensor muscles is required to keep the fingers away from their natural rest position. Therefore, the fingers slowly tend to restore a less fatiguing configuration. Conversely, the increased interdigit distance that occurred immediately after the removal of the continuous tactile feedback was an unpredicted outcome. This sudden opening motion evidently occurred in response to the removal of the continuous tactile feedback. The reasons of this opening motion are still to be entirely clarified, however, it may be a reflex response to the tactile event. Similar hand muscles reflexes were observed following pure cutaneous stimulation [36]. The motion occurred as soon as the contact force began to fade, which may lead us to exclude that the CNS produced it. Should it be the case, we would expect a longer latency between the stimulus and the motor response [19]. Given the challenging nature of the virtual reality task, as further reported by some participants, we can suppose that the participants co-contracted their muscles to keep the desired interdigit distance. This may have resulted in a large reflex amplitude, which is known to be proportional to the contraction force [37]. Possibly, the participants perceived the cube crushing, and as a reflex they suddenly opened the fingers to avoid breaking it. Oppositely, we could speculate that the participants subconsciously perceived the reduction of the grip force and consequently adapted the position of their fingers in the attempt to match it with the one that should correspond to the perceived amplitude of the reaction force. In the future, it will be of interest to evaluate different rates of stimulus removal, i.e. parameter λ of Eq. (2), to elucidate whether larger or smaller values produce different effects. Moreover, smoother functions should be tested in addition to the linear removal, such as a polynomial function that minimizes the mean-square jerk of movement [38].

## Conclusions

In conclusion, our study demonstrated that people can effectively incorporate continuous and transient tactile SSF in a task that required fine control of grasp. When the incidental feedback was unavailable, the continuous and the transient SSF delivered to the fingertips equally and significantly improved the manipulation performances. These results can be translated to the field of surgical robotics, where the improved manipulation performance and the great acceptability of the transient feedback could lead to higher success rate of the surgery and ultimately to a larger number of saved lives. In addition, the benefits of these results can be twofold in field of prosthetics. Firstly, considering that portable feedback systems for myoelectric prostheses are powered by portable batteries, removing the static feedback would allow considerable power saving with no performance drawbacks. Secondly, the recent opportunities brought by surgeries like TSR, represent excellent target applications of the outcomes of this study. Through this surgical technique, the fascicles of the nerves that carried most of the sensory contents are coapted to target cutaneous nerves of the residual limb to re-establish sensory maps of the hand, and reinnervated patients indeed experience hand sensations with broad spatial and force discrimination acuity [28]. The proposed sensory feedback strategy can therefore be leveraged to restore a physiologically appropriate haptic sensation to individuals who suffered the loss of a hand and make them feel their prosthetic fingers touching an object as naturally as they would do with their intact limb. Prostheses with integrated natural SSF might finally lead to a significant decline in their abandonment, which hitherto severely limited their widespread.

This work provided evidence of the feasibility of the transient feedback mechanism, but its full potential is still to be explored and more experiments are necessary to fully elucidate its effects. Different profiles of feedback removal should be investigated to optimize the procedure, as well as different tasks involving daily living or work activities. Finally, a larger pool of healthy participants could be enrolled to increase the statistical power of our study, and amputees treated with TSR should be involved in the experimental validation of our method, to ultimately demonstrate the potential of the proposed feedback modality in its envisioned application with neuroprostheses.

## Data Availability

The datasets used and analysed during the current study are available from the corresponding author on reasonable request.

## Abbreviations

CNS: Central nervous system
SSF: Supplementary sensory feedback
DESC: Discrete event-driven sensory feedback control
CoFB: Continuous feedback
NoFB: No feedback
TrFB: Transient feedback
TSR: Targeted Sensory Reinnervation
